# Double Consciousness

**Published:** 1845-09

**Authors:** 


*Double Consciousness.*—A. B. conceives that he is himself and another person at the same time; he acts as if this belief were sincere, and cannot divest himself of the conviction that in his own body are two minds or persons suggesting courses of conduct wisely opposed. He is certain that his original self, A. B., is a base abandoned scoundrel, tempting his other, or new, or better self, to whom it should be noted is attached the emphatic *Ego*, to commit crimes or acts of which he altogether disapproves. The second person of this duality repels, struggles with these abominable solicitations, such as that he should commit suicide, and loathes the tempter or first person. This struggle sometimes becomes real and visible, when the hands, acting under the will of No. 1, or the virtuous and opposing impulse, beat and bruise the legs, body, or head, which, it may be presumed, are supposed to belong to No. 2, the vicious or tempting impulse. The object of one is obviously to inflict pain upon the other. The blows are so severe as to leave marks for days, and when these are adverted to, the answer, is, as if from No. 2, "Don't justify him, he deserved it." Such conflicts generally occur during the night; the delusion appears to be the strongest at the time of awakening, and the interference of the Night Watch is required to part or pacify the combatants. The mind appears on these occasions to be so pre occupied by the delusion, as to confound the sensations communicated by these blows and to refer them to the body of another. In conversation with those around, A. B. speaks at one time as No. 1, at another as No. 2.

In the Second Case, the mind appears clear and coherent during the day, but insane or unsettled during the night, and when the patient is in a horizontal position. The healthy perceptions recognise the morbid ideas or dreams, for such it is probable they are, as appertaining to another state of being, and that they are the result of insanity. The patient is of cultivated mind, and able to analyse and describe his own feelings. He believes that he has two natures. For twelve hours he can laugh at the delusions of his companions and at his own; but during the night he supposes himself to be in a state of complete mesmerism; that he is mad and has the delusions of all the persons in the same gallery concentrated in himself; and that he is under the influence of supernatural power, which rules all his thoughts and actions, fills his mind with visions of the past and future, and enables him to accompany the Night Watch in his rounds, and to perform many inexplicable deeds, which are either repetitions of what he has done, or prefigure what he is still destined to do. He, the sane man, emphatically declares these to be the hallucinations of an insane man, who is himself, and yet different. Both of these manifestations of disease may be attributed to the abstruse but vain philosophical inquiries of the mind while in health.—*Ibid.*

